# Web-Based Decision Aid to Assist Help-Seeking Choices for Young People Who Self-Harm: Outcomes From a Randomized Controlled Feasibility Trial

**DOI:** 10.2196/mental.8098

**Published:** 2018-01-30

**Authors:** Sarah L Rowe, Krisna Patel, Rebecca S French, Claire Henderson, Dennis Ougrin, Mike Slade, Paul Moran

**Affiliations:** ^1^ Division of Psychiatry University College London London United Kingdom; ^2^ Department of Psychosis Institute of Psychiatry, Psychology and Neuroscience King’s College London London United Kingdom; ^3^ Department of Social and Environmental Health Research Faculty of Public Health & Policy London School of Hygiene and Tropical Medicine London United Kingdom; ^4^ Health Service and Population Research Department Institute of Psychiatry, Psychology and Neuroscience King’s College London London United Kingdom; ^5^ Child and Adolescent Psychiatry Department Institute of Psychiatry, Psychology and Neuroscience King’s College London London United Kingdom; ^6^ School of Health Sciences Institute of Mental Health University of Nottingham Nottingham United Kingdom; ^7^ Centre for Academic Mental Health School of Social & Community Medicine University of Bristol Bristol United Kingdom

**Keywords:** adolescent, self-harm, decision aid, intervention, schools, feasibility, randomized controlled trials, ethics

## Abstract

**Background:**

Adolescents who self-harm are often unsure how or where to get help. We developed a Web-based personalized decision aid (DA) designed to support young people in decision making about seeking help for their self-harm.

**Objective:**

The aim of this study was to evaluate the feasibility and acceptability of the DA intervention and the randomized controlled trial (RCT) in a school setting.

**Methods:**

We conducted a two-group, single blind, randomized controlled feasibility trial in a school setting. Participants aged 12 to 18 years who reported self-harm in the past 12 months were randomized to either a Web-based DA or to general information about mood and feelings. Feasibility of recruitment, randomization, and follow-up rates were assessed, as was acceptability of the intervention and study procedures. Descriptive data were collected on outcome measures examining decision making and help-seeking behavior. Qualitative interviews were conducted with young people, parents or carers, and staff and subjected to thematic analysis to explore their views of the DA and study processes.

**Results:**

Parental consent was a significant barrier to young people participating in the trial, with only 17.87% (208/1164) of parents or guardians who were contacted for consent responding to study invitations. Where parental consent was obtained, we were able to recruit 81.7% (170/208) of young people into the study. Of those young people screened, 13.5% (23/170) had self-harmed in the past year. Ten participants were randomized to receiving the DA, and 13 were randomized to the control group. Four-week follow-up assessments were completed with all participants. The DA had good acceptability, but qualitative interviews suggested that a DA that addressed broader mental health problems such as depression, anxiety, and self-harm may be more beneficial.

**Conclusions:**

A broad-based mental health DA addressing a wide range of psychosocial problems may be useful for young people. The requirement for parental consent is a key barrier to intervention research on self-harm in the school setting. Adaptations to the research design and the intervention are needed before generalizable research about DAs can be successfully conducted in a school setting.

**Trial Registration:**

International Standard Randomized Controlled Trial registry: ISRCTN11230559; http://www.isrctn.com/ISRCTN11230559 (Archived by WebCite at http://www.webcitation.org/6wqErsYWG)

## Introduction

### Background

Self-harm is common among young people, affecting about 1 in 10 people [1,2]. A past history of self-harm is also the strongest predictor of suicide, and so self-harm is a major public health concern [3].

Between a third and one half of young people who self-harm do not know where to seek help [4]. Given the reach of the Internet, Web-based approaches to supporting decision making may provide an important new way of providing decisional support.

Decision aids (DAs) are tools that assist the decision-making process by identifying the options available and the attributes associated with these options, as well as clarifying personal values and preferences [5,6]. They have been shown to increase knowledge, decrease decisional conflict, and encourage more active participation in decision making around matters relating to health [7]. DAs are more commonly used in adult populations, and little research has examined their potential utility within populations of young people or indeed, within the area of mental health [8,9]. We have developed (with the help of young people and clinicians) a Web-based personalized DA based on the principles of multi-criteria decision analysis (MCDA) [10] and designed to support young people in making help-seeking choices for their self-harm.

### Aims and Objectives

Our aim was to evaluate the feasibility and acceptability of the DA intervention and the randomized controlled trial (RCT) in a school setting. The objectives were as follows:

1. To assess recruitment or attrition rates, acceptability of randomization, data completeness, and feasibility of school-based sampling
2. To inform the design of an adequately powered, future effectiveness study through the reporting of descriptive data on candidate measures
3. To explore the views and experiences of the intervention and participating in the study with young people, staff, and parents

## Methods

### Trial Design

This was a two-group, parallel arm, single blind RCT. The trial protocol has been previously published [11], and in brief, outlines the development and piloting of the DA and plans for this feasibility study. We randomized school students who were screened for self-harming behavior to either receiving a DA (intervention group) or general information about mood and feelings (control group). Outcome measures using self-report questionnaires were completed at baseline (pre intervention), post intervention, and 4-week follow-up. Qualitative interviews were conducted with a subset of young people, staff, and parents to explore their views and experiences of the intervention and participating in the study.

### Participants

Inclusion criteria were young people aged 12 to 18 years attending the study site secondary school, with a basic proficiency in English language, and who had self-harmed in the past 12 months. Participants were excluded if they were lacking capacity to consent. This refers to young people who had cognitive or language difficulties that would preclude subjects being able to understand, retain, and weigh up information about the study and then communicate their decision regarding participation (ie, providing informed consent).

### Procedure

Potential participants were identified at a secondary school in an inner London borough. Parents or carers of students in the school were sent information by post, asking for their consent to invite their child to participate. This was followed by presentations at school assemblies (to students), school newsletters, circulation of a link to a podcast (audio recording) about the study, and a reminder email. Where consent was given, their child was invited to participate, and once consented, the participant completed a Web-based questionnaire at school (in their lunch break or after school) asking demographic questions (eg, age and gender), a short standardized questionnaire about their mood and feelings, and two questions about the occurrence of any self­-harm behavior in the previous year. If the participant did not report self­-harming in the previous year (including those who had never self-harmed and those who had self-harmed more than 12 months ago), the questionnaire ended, and they were given a paper copy of general information about feelings and emotions from the ChildLine website. For these participants, completion of the questionnaire was a one­time only occurrence that took approximately 5 to 10 min. All participants received a £5 voucher upon completion of the assessment to thank them for their time.

If the participant reported that they had self­-harmed in the previous 12 months, they completed baseline measures and were then randomized by a computer program to one of two groups: (1) a DA group who completed the DA and were then presented with help­-seeking options that were based on information they provided while using the DA. Once they completed the DA, they received a paper copy of information on how to access any of the help-seeking options that were listed in the DA and (2) a control group allocated to general information about feelings and emotions from the ChildLine website.

All randomized participants completed the measures before and after they went through the DA or control condition and at 4-week follow-up.

At the 4-week follow-up appointment, participants were invited for a qualitative interview to explore their views and experiences of the study procedures and for those randomized to the intervention, their views and experiences of the DA. Young people who had not indicated self-harm on the survey were also interviewed about their views of the study and potential utility of the DA for young people who self-harm. The sample was selected to include both males and females and a range of ages. All interviews were conducted during school hours in a private room on campus, and participants received a £10 Amazon voucher upon completion of the interview to thank them for their time.

Parents or carers of children attending the school (irrespective of whether their child had participated in the study) and teachers and pastoral staff were approached for a telephone interview to obtain their views on the intervention and recruitment into the feasibility trial. Consent for interview was obtained via email. Telephone interviews were conducted with parents or carers in a private room on the university site. All interviews were audio-recorded (with participant’s permission) and transcribed verbatim; details were anonymized to preserve participant identity.

### Ethics and Governance Approvals

This feasibility trial was approved by King’s College London College Research Ethics Committee; ref PNM/14/15-114, and the trial was registered with the International Standard Randomized Controlled Trial registry (ISRCTN11230559).

### Intervention Arm

The DA (called “My Self-Help Tool”) is based on the principles of MCDA and designed for young people to be used by themselves, to find out about different help-seeking options for self-harm (such as family, general practitioner, or telephone helpline). In addition to the sources of support, users were asked to identify help-seeking attributes that were of importance to them, ranging from confidentiality to other concerns (such as not wanting to be seen as attention seeking). They were then required to indicate the degree of importance they attached to each identified attribute according to the help-seeking options they had chosen, for example, weighting how important maintenance of confidentiality was to them. Once they had made their selections, a personalized rating and ranking of the help-seeking options was presented to them based on the information they had submitted (see Multimedia Appendix 1) [10,12].

### Control Arm

Participants in the control arm received general information on feelings and emotions from the ChildLine website. This information was displayed as a static (noninteractive) page in our questionnaire rather than a link that young people could use to connect to the ChildLine website. This comparison group was chosen to control for attention and time spent reviewing information. Those in the control arm were exposed to relevant static Web content but which did not involve decisional support.

### Safety Protocols

All participants who disclosed self-harm during the study (irrespective if it was in the past 12 months or more than 12 months ago) were referred to the school counselor to ensure they remained safe and were given appropriate support, as explained before enrollment in the study information sheet. Parents were also informed of this referral in accordance with school policy.

### Measures

#### Baseline Assessments: All Participants

The screening questionnaire comprised sociodemographics (gender, age, ethnicity, and living situation) and information about possible support networks (eg, siblings, boyfriend or girlfriend, or social worker), the Short Mood and Feelings Questionnaire (13-item self-rated measure of depressive symptoms with scores of 10 or greater indicating the likely presence of major depression) [13], and two questions on self-harming behavior: (1) “Have you ever deliberately tried to harm yourself (such as cut yourself or taken an overdose)?” and (2) “When was the last time you tried to harm yourself?” [14].

#### Decision Aid Group and Control Intervention

1. Stage of decision making scale: This scale measures the individual’s readiness to engage in decision making [15]. It consists of one item with six response options anchored at 1 (haven’t started to think about the choices) and 6 (have already made a decision and am unlikely to change my mind).
2. General Help-Seeking Questionnaire (GHSQ, intentions): This assesses future help-seeking intentions and recent and past help-seeking experiences [16]. It uses a 7-point Likert scale ranging from 1 (extremely unlikely) to 7 (extremely likely) for each help source option. Higher scores indicated higher intentions.
3. Questionnaire on anticipated discrimination: This 14-item questionnaire measures the extent to which people anticipate personally experiencing discrimination in key life domains as a result of mental health problems [17]. It uses a 4-point Likert scale ranging from 0=strongly disagree to 3=strongly agree. We used five items from this measure that are relevant to our study population (adolescents).
4. Decisional Conflict Scale (DCS): The decisional conflict scales measure personal perceptions of (1) Uncertainty in choosing between options, (2) Modifiable factors contributing to uncertainty, and (3) Effective decision making. The 16-item version of the scale was used [18]; each item is rated on a 5-point Likert scale ranging from 0=strongly agree to 4=strongly disagree. A total score and 5 subscores (uncertainty subscore, informed subscore, vales clarity subscore, support subscore, and effective decision subscore) are generated. Scores exceeding 37.5 are associated with decisional delay or feeling unsure about implementation. We asked questions pertaining to the support and uncertainty subscales only.
5. Questions on the DA (only completed by those allocated to the DA group): Participants were asked to answer questions about (1) whether they would follow the “recommended” option, (2) whether the use of the DA has changed any of their perceptions or feelings about their help-seeking options, (3) whether there is anything we could do to improve the DA, and (4) whether or not they would recommend the DA to other young people who have self-harmed.

The support and uncertainty subscales from the DCS and the Stage of Decision Making scale were repeated immediately after completing the DA. All measures were repeated at 4 weeks, with the only change being to the GHSQ, which asked about actual help-seeking behavior in the past 4 weeks.

Once this assessment was completed, all participants (and a small sample of nontrial participants) were invited to participate in a qualitative interview, which sought to explore factors relating to participation in the study and for intervention participants, views, and experiences of the DA (eg, how, if at all, the DA prompted help-seeking behavior).

#### Sample Size

We undertook a power calculation to give us a target number to aim toward. For continuous outcomes relating to decision making and empowerment, a sample size of 60 (30 randomized to the DA and 30 randomized to the control condition) would detect a standardized effect size of 0.75, with 80% power [19]. To obtain a sample of 60 young people reporting self-harm to be randomized, we needed to recruit and screen 600 pupils based on a 10% prevalence rate [2].

#### Randomization and Blinding

Following consent and baseline assessment, participants who were randomized into the intervention and control groups (ie, those who had self-harmed in the past year) were placed into one of eight trial strata (all boys were grouped into a single stratum, and girls were grouped into seven age strata). We stratified randomization by gender because self-harm typically occurs more frequently in female compared with male adolescents [20]. Each stratum was allocated using a random permuted block algorithm, with a block size of four. Appropriate locks were in place to ensure that randomization tokens could not be used multiple times or skipped over. Research staff were blinded to allocation of the intervention or control arms at post intervention and follow-up.

#### Statistical Analysis Plan

In keeping with recommendations for small-scale feasibility trials, the analysis focused on feasibility of scaling up to a full-scale RCT [21]. This consisted of the following:

1. To determine feasibility of recruitment to the study, parental and student invitation and consent rates were documented, and the number of young people meeting inclusion or exclusion criteria were examined. Details of those who declined to be randomized and an option for their reason for refusing were recorded.
2. Treatment acceptability was assessed by the proportion of participants who refused to use the DA. Retention up until 4-week follow-up was examined.
3. Feasibility of the research protocol was assessed by the proportion of participants failing to adhere to the full research protocol, the burden of which will be similar to that which could be expected in a full study. The target collection of complete data was 90% of all participants recruited.
4. Exploratory findings were conducted on outcome measures, and we used a CI approach to assist the justification for proceeding to a future trial. A linear regression with the treatment group contrast was used to assess the difference between groups, adjusted for baseline differences. A linear regression with a single binary explanatory variable is equivalent to a *t* test. It was chosen instead of a *t* test because it is not possible to perform a *t* test with more than one explanatory variable, so we would not have been able to adjust for baseline using a *t* test. Effect sizes for partial eta squared are larger values than eta-squared, ranging from 0.01 (small), 0.09 (medium), and 0.25 (large). All analyses were performed with Statistical Package for the Social Sciences (SPSS) versions 22.0 and 24.0 (IBM Corp) for Windows.

#### Qualitative Analysis

Thematic analysis [22] was used for this study. After initial familiarization with data from the first five transcripts, KP and RF generated a preliminary coding framework. Primary analysis was undertaken by one author (KP) using Nvivo10 (QSR International) [23]. KP coded all the interviews using the agreed framework with themes and subthemes identified, along with deviant and atypical cases. Discussions were held to ensure themes were adequately reflected in the raw data [11].

## Results

### Quantitative Findings

#### Feasibility of Recruitment and Consent Rates

**Figure 1:**
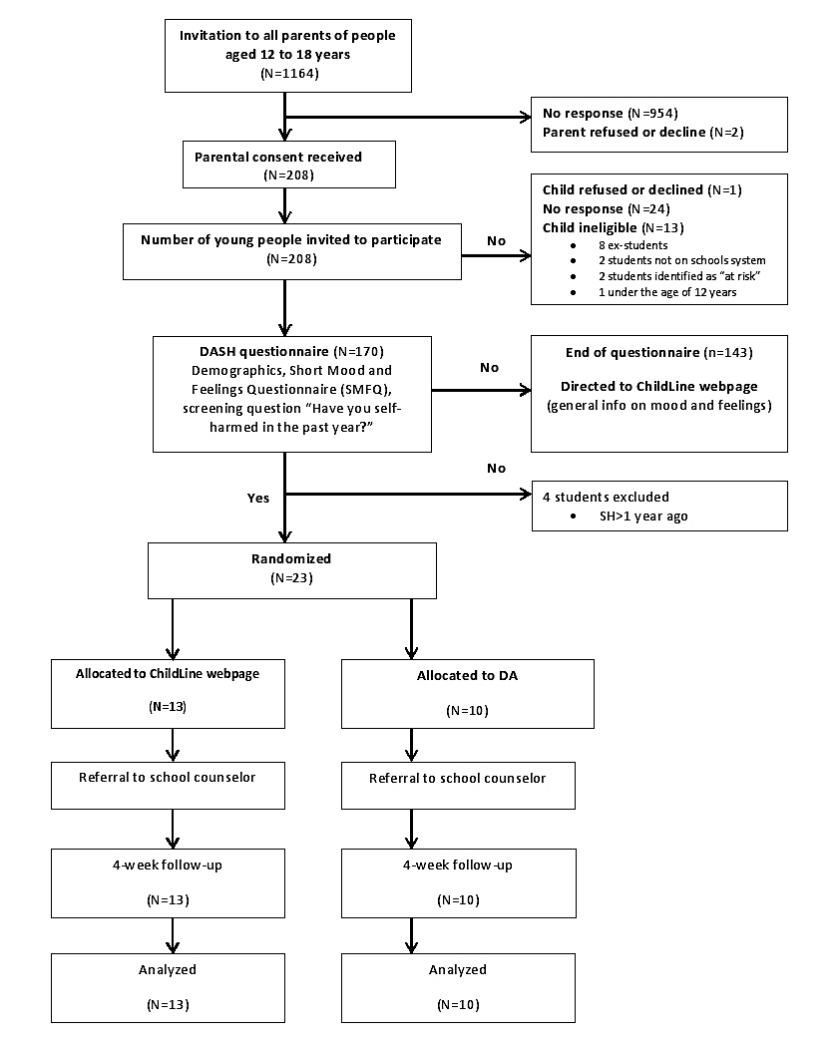
Flowchart of the trial design. DA: decision aid.

Recruitment is shown in Figure 1. A total of 208 parents or guardians gave consent (17.87%, 208/1164). Attempts to contact the 208 young people resulted in 170 (81.7%) participants recruited over 10 months (October 2015-July 2016). No young people were excluded on the grounds of lack of capacity to consent.

#### Feasibility of Randomization Procedures, Response Rates, and Follow-Up Rates

Of the 170 young people who were screened using the Web-based questionnaire, 23 (13.5%) reported self-harming behavior in the past year (and 5 reported self-harm occurring over a year ago). None of the 23 eligible participants declined to be randomized (intervention n=10, control group n=13). Final follow-up was completed in August 2016. No baseline, postintervention, or 4-week follow-up data were missing.

#### Baseline Characteristics and Previous Help-Seeking for Self-Harm

Demographic data for the sample are displayed in Table 1.

**Table 1:** Descriptive statistics for study sample.

Demographics		Decision aid (N=10)	Control (N=13)	Not randomized (N=147)
**Gender, n (%)**				
	Female	6 (60)	8 (62)	77 (52.3)
	Male	4 (40)	5 (38)	70 (47.6)
**Ethnicity, n (%)**				
	White	10 (100)	11 (84)	110 (74.8)
	Mixed or multiple	0 (0)	1 (8)	14 (9.5)
	Asian or Asian British	0 (0)	0 (0)	5 (3.4)
	Black or African or Caribbean or black British	0 (0)	0 (0)	14 (9.5)
	Other	0 (0)	1 (8)	4 (2.7)
**Age (years)**				
	12-15	8 (80)	12 (92)	138 (93.8)
	16-18	2 (20)	1 (8)	32 (21.8)
**Short Mood and Feelings Questionnaire total score, mean (SD)**				
	0-10	4 (40)	6 (46)	133 (90.5)
	11-26 (depressive symptoms)	6 (60)	7 (54)	14 (9.5)

**Table 2:** Linear regression of outcome measures.

Outcome measures		Decision aid	Control	Regression		
		Mean (SD)	Mean (SD)	Group difference (95% CI)^a^	*t, P* values	Effect size (η^2^)^b^
**Stage of decision making**						
	Preintervention	3.7 (2.0)	3.1 (2.1)			
	Postintervention	4.0 (1.6)	4.0 (1.7)	−0.4 (−1.4 to 0.8)	−0.68, .51	0.02
	4-week follow-up	3.5 (3.5)	4.8 (1.6)	−1.4 (−3.0 to 0.3)	−0.17, .10	0.13
**DCS^c^uncertainty subscale**						
	Preintervention	31.7 (32.8)	35.9 (33.9)			
	Postintervention	31.7 (33.7)	37.2 (35.5)	−1.3 (−11.1 to 8.4)	−0.29, .78	0.00
	4-week follow-up	40.0 (42.5)	30.8 (27.9)	11.1 (−14.3 to 37.7)	0.94, .36	0.04
**DCS support subscale**						
	Preintervention	15.0 (18.3)	19.2 (23.4)			
	Postintervention	15.0 (24.2)	11.5 (19.7)	6.8 (−5.4 to 19.0)	1.17, .26	0.06
	4-week follow-up	21.7 (26.1)	15.4 (18.6)	5.7 (−14.1 to 25.6)	0.60, .55	0.02
**Questionnaire on anticipated discrimination**						
	Preintervention	4.5 (2.7)	5.4 (2.2)			
	4-week follow-up	6.1 (3.6)	4.4 (2.1)	0.4 (−0.8 to 0.9)	1.76, .10	0.13
**GHSQ^d^(intentional help-seeking)**						
	Preintervention	36.4 (8.4)	45.8 (8.9)	9.1 (−16.7 to −1.4)	2.47, .02	0.23
**GHSQ (actual help-seeking)**						
	4-week follow-up	1.6 (1.8)	2.2 (1.9)	0.6 (−2.3 to 1.0)	0.80, .44	0.03

^a^Adjusted for baseline scores.

^b^Partial eta squared.

^c^DCS: Decisional Conflict Scale.

^d^GHSQ: General Help-Seeking Questionnaire.

Of the 23 who had self-harmed in the past year, 6 (26%) had never disclosed their self-harm, and of the 17 that had previously disclosed, 9 (53%) had found the support helpful. Friends and family were the most common sources of disclosure (n=11).

Descriptive data on outcome measures are presented in Table 2. There was no difference is completion rates and minimal difference in the administration time of the measures. CIs around the coefficient estimates were very wide indicating, as anticipated, that the study was underpowered to detect significant differences.

#### Acceptability of the Intervention

All 10 young people (100%, 10/10) randomized to the intervention stated (1) they would follow the DA advice, (2) it had changed their attitude regarding help-seeking behavior, and (3) they would recommend the DA to others that are self-harming. No adverse events were reported as a result of using the DA.

### Qualitative Findings

#### Participant Characteristics

A total of 14 young people were interviewed, comprising 9 trial participants (8 DA, 1 control) and 5 not reporting self-harming behavior (Table 3). Interviews lasted between 8 to 45 min. Three members of school staff (the school counselor, deputy head teacher and safeguarding officer, and a teacher) and 5 parents or carers were interviewed (all of whom had consented to their child’s participation), with interviews lasting between 8 to 15 min.

The major themes were (1) Reason for participation, (2) Views and experiences of the intervention, and (3) Feasibility of delivering the intervention and conducting an RCT in a school setting.

#### Reasons for Participation

Study participation was discussed in the context of facilitators and barriers, namely, encouragement from others, a financial incentive, and stigma. Support from peers, the school counselor, and parents or carers were reported to be key factors in the decision to participate, as was the financial reward.

Anonymity, confidentiality, trust, and judgment were discussed independently and in relation to each other and appeared to play a key role in the young person’s decision to participate in this research and disclose self-harming behavior. For example, confidentiality was referred to as both a facilitator and barrier to study participation and disclosure of self-harm; some students were not concerned about confidentiality and saw participation as an opportunity to get support; others felt that the possibility of other adults and young people discovering participation prevented involvement and disclosure, as illustrated in the following quote:

Fear of all the...fear of parents finding out and fear of the school knowing, just walking around the school knowing that all of headship team know you’ve done something, just...and you, kind of, give...it’s the stigma around it, really.

Student 11, nonself-harm

Many young people interviewed had chosen not to discuss self-harm before study participation, with several reasons reported including lack of time, embarrassment, and not feeling ready for disclosure and possible professional support and intervention. Timing in relation to the young person being ready to seek support was a key factor for their decision on whether or not to take part in the study, as illustrated in the following quote:

...it (self-harming) just started escalating and then...I think the survey came at the right time for me because otherwise something, it could have gone anywhere.

Student 5, self-harm

Both those who had indicated and those who had not indicated self-harm reported that belonging to a larger group of participants also facilitated the decision to participate, and this was also discussed in the context of helping others who may have been going through the same thing as them, as illustrated in the following quote:

I know people that do self-harm, and I think it might be helpful for them, because they might get the support that they need through it.

Student 14, nonself-harm

Wider coverage of the study around the school and in the community to raise awareness of the research but also the prevalence of self-harm and mental health issues in young people was recommended to encourage participation and disclosure.

#### Views and Experiences of the Intervention

Young people reported a preference for a computer-based intervention and found the DA to be “*quick*” and “*easy to use.*” It was largely described as clear and comprehensive, although some students did report that it could be better tailored for younger students in terms of phrasing of language and interactivity. There were several other recommendations on how the intervention could be improved.

These included changing some aspects of the interface and language of the questions to make it clearer, broadening the scope of the DA so it was relevant to other mental health problems such as depression and anxiety, making the DA more widely available (to the general public), providing more information about the help-seeking options (eg, “what does a psychologist do?”), and embedding the tool within a general mental well-being context (eg, providing psychoeducation about mental health issues to young people and how to manage distress) so it could potentially reduce the stigma and isolation around self-harm and mental illness.

**Table 3:** Participant demographics and characteristics for qualitative interviews.

Student ID	Gender, female (F) or male (M)	Age range (years)	Self-harm (SH)^a^ or nonself-harm (non-SH)^b^
1	M	12-15	SH
2	F	12-15	SH
3	F	12-15	SH
4	F	12-15	SH
5	F	12-15	SH
6	M	12-15	SH
7	M	12-15	SH
8	M	16-18	SH
9	F	12-15	SH
10	F	12-15	Non-SH
11	M	16-18	Non-SH
12	F	12-15	Non-SH
13	M	12-15	Non-SH
14	M	12-15	Non-SH

^a^Randomized.

^b^Not randomized.

For those who had self-harmed, the intervention (or participating in the study) provided the space to think about their behavior and the opportunity to open a dialogue about it, and for those who hadn’t, it raised awareness of self-harming behavior and potential sources of support, as illustrated in the following quotes:

It kind of made me, that’s kind of when I stopped feeling suicidal really as much as I did, but I haven’t stopped completely

Student 9, self-harm

Well it definitely made me think, like, about the situations and self-harm a lot more. Like, I think if I never came to the study, I don’t think that would ever have crossed my mind, or anything

Student 6, self-harm

For those who had reported self-harm and been randomized to the DA, this was also in the context of identifying different sources of support previously unknown to them, reducing the potential shame and judgment associated with disclosing behavior and seeking professional support. Participation also acted as a signpost to other potentially useful sources such as telephone helplines and online sources for some young people.

Postintervention outcomes were discussed in the context of the participant’s survey responses and whether the DA recommendation was what they were expecting and if they felt they were able to follow the advice of the DA. Although some young people were expecting the outcome, others reported being surprised by the recommended option, as illustrated in the following quotes:

Yes. It was useful because I think the GP came quite high up, which is...No, because I didn’t really know about, that you could get, like, help from the GP. So I thought that was quite interesting. I, you know, if anything happens I can just pop down the road to the GP practice

Student 7, self-harm

Because like, I wouldn’t really talk to my mum for example, and then when it came up on that I was like, really?

Student 2, self-harm

Reports were mixed on whether participants chose to follow the advice of the DA (this was particularly the case if a professional help-seeking option was recommended), which is contradictory to what they indicated in the free-text of the survey, post intervention (where they had all indicated they would follow the advice of the DA), as illustrated in the following quote:

I talked to my very close friends. I didn’t get to see my GP, but I talked to my mum and my dad about it.

Student 1, self-harm

Some young people described experiencing some cognitive-affective and post intervention behavioral change as a result of completing the DA, including increased awareness of different sources of support previously unknown to them, empowerment, self-reflection, reducing the potential shame and judgment associated with disclosing behavior, and help-seeking. However, others did not feel it had changed anything specific, as illustrated in the following quote:

What have I done? I’ve watched like, videos on YouTube of how to like, calm yourself down. So like crushing ice and cuddling a soft toy and things

Student 2, self-harm

Seeking professional support was largely discussed in reference to meeting the school counselor as a part of the safety protocol, which helped some young people identify alternative coping strategies in response to distress, as illustrated in the following quote:

And it’s been really interesting all this, and I’ve learnt about how to get help for self-harm, what to do. It’s also tied me into the school counsellor which is really helpful for me. I think that was more beneficial because I could have easily just slipped back and things have gone worse again, but it’s good that you guys are keeping tabs on me

Student 7, self-harm

#### Feasibility of Delivering the Intervention and Conducting a Randomized Controlled Trial in a School Setting

The majority of participants reported that recruitment, completing the survey, and follow-up were straightforward and short processes and enjoyed participating in topical research.

The study, which required a room with a computer and Internet connection to complete the questionnaire and intervention, was not found to be too burdensome on resources. Furthermore, the school counselor, to whom all the randomized students were referred, did not feel that the study placed a significant burden on their workload. A member of staff reported that the schools participation in the study showed its commitment to addressing issues around mental health, as illustrated in the following quote:

I think that then that’s highlighted issues that, kind of, maybe needed to be talked about more to do with self-harm, anxiety, mental health in general, so I think that’s been really a positive part of it, and promoting...yes, promoting, kind of, awareness of...

School staff member

Parents also thought that the school setting was an appropriate place to deliver the intervention, as illustrated in the following quote:

I mean, I think, yes, all young people on the whole are in school and not many go to seek help and you kind of have to make it easy for people to, you know, get support where they are and where they’re spending time and where there is, you know, some kind of safe environment around them

Parent or carer

Despite these comments, the response from parents or carers giving consent for us to invite their children to this study was low. Interviewed parents or carers thought that this may be because some parents believed the topic of self-harm was not relevant to their children; staff speculated that this may have been because of anxiety around “contagion” of self-harm, as illustrated in the following quote:

I think it was mainly just around the anxiety and then the idea that the, sort of, contagion effect that, oh, yes, if I say yes to this, then it’s going to actually make it happen or make my child feel this, this, or make them self-harm or maybe if they’re a part of it, then them and their friends, it’ll, sort of, spread, but I think mainly around anxiety

School staff member

Areas for improvement (and so it might facilitate better engagement from parents) were similar to the recommendations given by the young people participating in these interviews. An additional suggestion was providing resources to parents or carers on increasing resilience and identifying or managing self-harming behavior in their children.

## Discussion

### Principal Findings

Undertaking self-harm research in a school setting is challenging. Although we recruited 81.7% (170/208) of young people into the study whose parents had given consent (13.5% of whom had self-harmed in the past year), fewer than 1 in 5 parents consented to us contacting their child. We were able to follow up all participants 4 weeks post intervention with no dropouts or missing data. The impact on school resources (eg, the school counselor’s workload) was minimal, and the school reported that the study promoted the awareness of mental health issues and services available to students.

The sample size was too small and CI’s too wide to make assumptions about the required sample size for a larger RCT. Findings were inconsistent between the survey and interviews on whether or not young people would follow the help-seeking recommendations from the DA, particularly if a professional option was recommended. This suggests the possibility of acquiescence bias.

Overall, those that were randomized to the DA found the intervention acceptable and would recommend it to other people that were self-harming. There were no differences in the measures, and all would be potentially appropriate for use in a larger future trial.

### Strengths and Limitations

In addition to underrecruitment and small sample size, this study had several other limitations. Our safeguarding procedures may have reduced participation in the study and indeed enforced a help-seeking option (ie, referral to a school counselor). This was included in the protocol to maintain the safety of all trial participants who were considered to be a particularly vulnerable group on account of their age and the occurrence of their self-harm. However, the inclusion of this procedure may have dissuaded young people from answering the self-harm screening question honestly if they did not want the school or their parents to find out about their behavior. As all trial participants were referred to the school counselor, any impact of the DA may have been obscured.

We did not control for the effects of simply interacting with the Internet. Participants in the intervention group interacted with live content on a Web page, whereas those in the control condition interacted with static content. Research suggests that interaction with technologies can affect group decision making [24]. Further investigation is necessary to explore whether this is also the case at an individual level. The 4-week follow-up was short and did not allow sufficient time to assess change in decision making or help-seeking behavior. We collected limited information on self-harm behavior, such as frequency, type, and severity, and we were unable to obtain any qualitative information from nonconsenting parents.

There were a number of strengths in this feasibility trial. First, rates of self-harm were around those expected at 13.5% [2], suggesting that any negative impact of the safeguarding protocol was minimal within the sample recruited. Second, by indirectly including the school counselor as part of the intervention, we created a safe, pragmatic approach to the implementation of the DA in a school setting. E-mental health interventions have been shown to be effective tools; however, they often suffer from poor engagement from service users [25]. It has been suggested that including a face-to-face element can improve adherence and outcomes [26,27]. Third, qualitative interviews with students (both randomized and nonrandomized), parents or carers, and staff provided a deeper understanding of their views and experience of the DA and the process or implementation of conducting an RCT in a school setting. This helps us plan further developmental work to the DA and changes in future study design.

### Implications for Future Research

Although it is best practice to obtain parental consent (“active consent”) before the involvement of young people’s participation in a study, previous research shows that this lowers response rates by 40% to 67% compared with passive consent (eg, opt out) and results in decreased participation in school surveys by at-risk groups [28]. This raises several important ethical issues: (1) is the topic of self-harm one for which mature and competent young people should be able to give consent without prior consent from their parents? (2) should research in the area of self-harm be automatically categorized as above the threshold of minimal-risk research? and (3) is the importance of the topic sufficiently high and the research so hampered by the current ethical safeguards that more harm is caused by the safeguards (as they effectively make it impossible to create an evidence base for this public health problem) than they benefit?

The mature-minor principle (“Gillick competency”) acknowledged by the United Kingdom and the United States recognizes children’s rights to consent to their own medical treatment without parental consent, if they have been deemed competent, based on their level of maturity rather than their age [29]. This extends to a research context whereby the Council for International Organizations of Medical Sciences refers to the waiving of parental consent in studies exploring adolescent’s beliefs and sexual behaviors or recreational drug use if it puts young people at risk of questioning or intimidation by their parents [30,31]. However, there is the need to balance the right to autonomy and access to participating in research with the risk of harm. In the case of self-harm research, this is usually considered above the threshold of minimal-risk; however, there is a lack of evidence supporting possible adverse effects of intervention studies on suicidal behavior [32,33]. Previous research suggests school-based interventions targeting suicidal behavior do not pose a risk of harm and may promote mental health awareness and reduce suicidal behaviors [34,35].

The complex issues around youth health research ethics are ongoing [31,36] and evident in the limitations of our feasibility trial. Research ethics committees may need to show greater flexibility in their interpretation of the guidelines around the necessity of parental consent for young people participating in self-harm research. Without this, we fear that research in the area will continue to be hampered by low response rates.

Going forward, further development of the DA may benefit from considering models of decision making within adolescent populations. Models that theoretically underpin the design of DAs have been confined to clinical decisions and settings and are largely based on adults; there is some evidence to suggest differences in decision-making processes between adolescents and adults [37]. The utility of the DA could be enhanced by including self-harm ideation. Adolescents who have thoughts of self-harm but have not engaged in self-harming behavior may be unaware of the benefits of seeking help, and the DA could potentially provide them with useful information on available support. Finally, if we are able to show proof of concept for a DA regarding help-seeking for self-harm, we can explore its applicability for other mental disorders.

## Appendix

Multimedia Appendix 1 Demonstration of decision-aid "My Self-Help Tool".

Multimedia Appendix 2 CONSORT‐EHEALTH checklist (V 1.6.1).

## Abbreviations

DA: decision aid
DCS: Decisional Conflict Scale
GHSQ: General Help-Seeking Questionnaire
MCDA: multi-criteria decision analysis
RCT: randomized controlled trial
